# Pairwise and higher-order correlations among drug-resistance mutations in HIV-1 subtype B protease

**DOI:** 10.1186/1471-2105-10-S8-S10

**Published:** 2009-08-27

**Authors:** Omar Haq, Ronald M Levy, Alexandre V Morozov, Michael Andrec

**Affiliations:** 1 BioMaPS Institute for Quantitative Biology, Rutgers, the State University of New Jersey, 610 Taylor Road, Piscataway NJ 08854, USA; 2 Department of Chemistry and Chemical Biology, Rutgers, the State University of New Jersey, 610 Taylor Road, Piscataway NJ 08854, USA; 3 Department of Physics and Astronomy, Rutgers, the State University of New Jersey, 136 Frelinghuysen Road, Piscataway NJ 08854, USA

## Abstract

**Background:**

The reaction of HIV protease to inhibitor therapy is characterized by the emergence of complex mutational patterns which confer drug resistance. The response of HIV protease to drugs often involves both primary mutations that directly inhibit the action of the drug, and a host of accessory resistance mutations that may occur far from the active site but may contribute to restoring the fitness or stability of the enzyme. Here we develop a probabilistic approach based on connected information that allows us to study residue, pair level and higher-order correlations within the same framework.

**Results:**

We apply our methodology to a database of approximately 13,000 sequences which have been annotated by the treatment history of the patients from which the samples were obtained. We show that including pair interactions is essential for agreement with the mutational data, since neglect of these interactions results in order-of-magnitude errors in the probabilities of the simultaneous occurence of many mutations. The magnitude of these pair correlations changes dramatically between sequences obtained from patients that were or were not exposed to drugs. Higher-order effects make a contribution of as much as 10% for residues taken three at a time, but increase to more than twice that for 10 to 15-residue groups. The sequence data is insufficient to determine the higher-order effects for larger groups. We find that higher-order interactions have a significant effect on the predicted frequencies of sequences with large numbers of mutations. While relatively rare, such sequences are more prevalent after multi-drug therapy. The relative importance of these higher-order interactions increases with the number of drugs the patient had been exposed to.

**Conclusion:**

Correlations are critical for the understanding of mutation patterns in HIV protease. Pair interactions have substantial qualitative effects, while higher-order interactions are individually smaller but may have a collective effect. Together they lead to correlations which could have an important impact on the dynamics of the evolution of cross-resistance, by allowing the virus to pass through otherwise unlikely mutational states. These findings also indicate that pairwise and possibly higher-order effects should be included in the models of protein evolution, instead of assuming that all residues mutate independently of one another.

## Background

The protease enzyme coded for by the *pol *gene of the Human Immunodeficiency Virus HIV-1 plays a critical role in the reproduction of the virus by cleaving the GAG precursor protein in a sequence-specific manner into its functional form, and as such, is a key target of several families of commonly used drugs used to control HIV infection [1]. Unfortunately, the virus has been able to evolve resistance to many of these drugs, in part due to the high mutation rates in the HIV genome [2]. The patterns of mutations in protease are complex, involving multiple key primary mutations that inhibit the action of drugs and a host of accessory mutations that can modulate the enzyme's stability or activity or otherwise enhance the fitness of the virus. It is now understood that these mutations do not occur independently of each other, but instead are correlated, resulting in complex patterns of co-evolving mutations [3-7].

Previous studies have mostly focused on correlations between mutations in the HIV protease gene at the pair level [3,5-7]. However, recognition that the observed mutations may also be involved in higher-order interactions has led to a few studies in which correlated pairs of mutations are grouped using tools such as multidimensional scaling [3,6], Bayesian networks [8], networks defined by patterns of conditional selection pressure [5], and clustering [9,10]. The underlying assumption is that understanding higher-order interactions is important for a complete understanding of the evolution of resistance in HIV protease.

In this paper, we investigate correlations among HIV protease mutations at and beyond the pair level, and the impact of drug treatment on the nature of those correlations. We only consider the presence or absence of a non-synonymous mutation relative to a defined wild-type sequence, and not the precise base or amino acid substitution which has occurred. We develop a hierarchy of probabilistic log-linear models [11] that can in principle describe residue interactions of arbitrary order, and use those to analyze HIV protease sequence data obtained from patient cohorts with varying protease inhibitor (PI) treatment histories.

We use "connected information" [12] to quantify inter-residue interactions at the triplet and higher level. Unlike the Bayesian network approach [13], the information-theoretic methodology allows us to distinguish intrinsic three-body effects from the cases in which correlations between three random variables can be attributed mostly to pairwise interactions. The connected information viewpoint of higher order correlation has not been previously used in the analysis of mutational patterns in HIV protease, although it has been employed in a much more limited analysis of the V3 loop of the HIV envelope protein [14], and log-linear models have been used to study protein-protein interactions [15]. We find that pairwise interactions are necessary to achieve even qualitative agreement with the mutational data, while higher order interactions play an important role in predicting how frequently sequences with several mutations appear in the database. Simultaneous appearance of multiple mutations may play an important role in the phenomenon of multiple- or cross-resistance of the viral protease.

## Results

### Increased mutation frequencies under drug exposure

**Figure 1:**
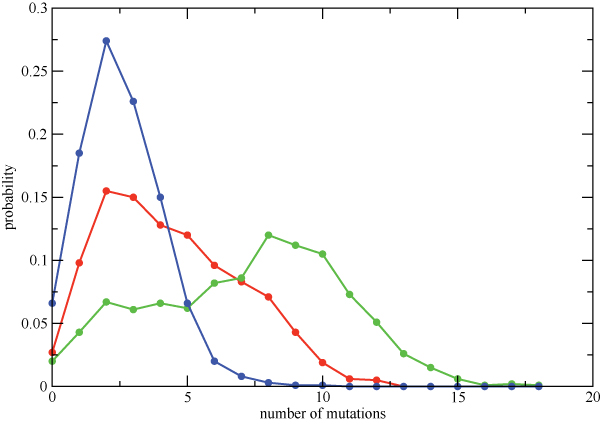
**Probability distributions for the total number of mutations among the 41 drug-associated residues in HIV protease for sequences obtained from the PI0 (blue), PI1 (red), and PI2+ (green) cohorts**.

As has been previously observed [3], we find that the overall number of mutations seen in HIV protease increases significantly with the number of PIs that the patient has been exposed to. This is seen in Figure 1, where we show the distribution of the number of mutations at drug-associated positions for sequences isolated from the drug-naive cohort (PI0), from a PI monotherapy cohort (PI1), and from a cohort treated with 2 or more PIs (PI2+), as estimated from the database described in the Methods. For some residue positions, the increase in the mutation frequency between the PI0 and PI2+ cohorts is nearly eightfold, while other positions show no discernible change (Additional File 1). This observed increase in mutation frequencies at drug-associated sites is largely responsible for the shift in the distribution shown in Figure 1.

It is of interest to ask which amino acid positions exhibit elevated mutation frequencies under drug treatment. Mutations at many of these positions are associated with decreased HIV-1 inhibitor susceptibility, and it is useful to classify mutations as belonging to "primary" vs "accessory" resistance classes. The terms "secondary" and "compensatory" have also been used as synonyms for "accessory". The specific criteria for such a classification are *ad hoc *in nature, but have generally been defined as follows.

Primary mutations are usually selected first in the presence of the drug and confer resistance, even when present as single point mutations [16,17]. They can be structurally important, e.g. situated near the enzymatic active site, in which case their effect on inhibitor binding can be rationalized due to their physical proximity to the inhibitor [18]. In the case of protease, however, there are exceptions, as some mutations (such as positions 54, 76, 88 and 90) are situated far from the active site or have no direct contacts with the substrate, yet still reduce drug susceptibility [16]. The mechanism of action of these mutations is not clearly understood [19].

Accessory mutations confer resistance only when present with additional primary or accessory drug-resistance mutations and have little or no effect on inhibitor susceptibility on their own. Some of these mutations occur in the absence of drug treatment, but their frequency of occurrence is observed to increase in treated patients. Accessory mutations may "rescue" possible losses of activity or stability in the enzyme that may have been caused by a destabilizing primary resistance mutation, and therefore may have a compensatory function in restoring viral fitness [20].

We make use of a primary and accessory classification scheme based on the work of Shafer et al. [21-23,17]. We define 17 primary drug resistance positions (residues 23, 24, 30, 32, 33, 46, 47, 48, 50, 53, 54, 73, 76, 82, 84, 88, 90) and 24 accessory drug resistance positions (residues 10, 11, 13, 20, 34, 35, 36, 43, 45, 55, 58, 60, 63, 71, 74, 75, 77, 79, 83, 85, 89, 91, 93, 95). The remaining positions are polymorphic mutations not associated with drug resistance or are conserved sites [24,3,21,26,23].

**Figure 2:**
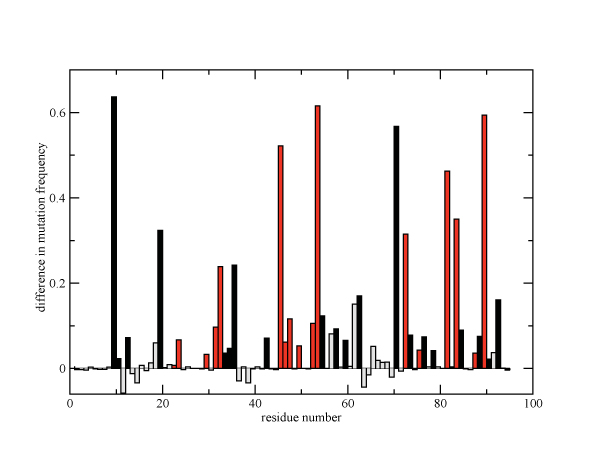
**Difference in the frequency of mutated residues between patients treated with 5PIs and PI-naive patients for all positions in HIV protease.  **Red bars correspond to primary drug resistnce positions, black bars are accessory drug resistance positions, and grey bars are positions not associated with drug resistance**.**

As seen in Figure 2, the positions which exhibit the most elevated mutation frequencies in PI2+ relative to PI0 sequences are for the most part primary and accessory drug resistance positions. It is interesting to note that sequences bearing mutations at any one of the 6 residues with the most elevated mutation frequencies (10, 46, 54, 71, 82 and 90) are resistant to most of the current PIs, including amprenavir (APV), indinavir (IDV), nelfinavir (NFV), ritonavir (RTV), saquinavir (SQV), atazanavir (ATV), and lopinavir (LPV). For instance, according to the Stanford HIV database [16], the common mutations at these residues (L10I, M46I, I54V, A71V, V82A and L90M) are associated with six or all seven of the drugs mentioned above. On the other hand, primary drug resistance positions that show the least amount of change in their mutation frequency (23, 30, 76 and 88) are generally inhibitor-specific. For example, D30N is associated only with NFV treatment, L23I and L76V are specific to two drugs each (NFV and SQV, and IDV and LPV, respectively), while N88D is specific to IDV, SQV and NFV treatment. Thus, positions that provide drug resistance to multiple inhibitors are more frequently mutated in the PI2+ cohort than positions that are specific to a small number of protease inhibitors. Upon examination of 278 ligand-bound crystal structures of HIV-1 protease, no heavy atom of residues 10, 46, 54, 71, or 90 is ever within 3.4 Å of any ligand bound at the cleft (data not shown). The atoms of residue 82, however, do contact ligands in 54% of the crystal structures examined.

### Exhaustive analysis of residue pairs

**Figure 3:**
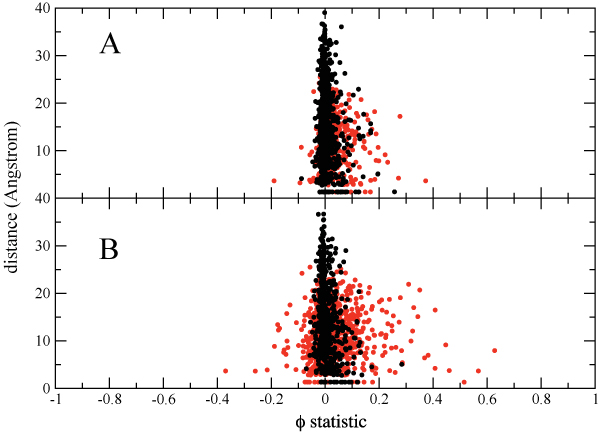
**Scatterplot of distance vs *ϕ *statistic for drug associated positions in red and non-drug associated positions in black for the PI0 (A) and PI2+ (B) cohorts**. The value along the y-axis is the closest distance between any two heavy atoms of the two residues based on the crystal structure of wild type protease (PDB ID 1PRO).

We begin by investigating pair correlations and their association with the structure of HIV protease. Several groups have studied pair correlations as a means of identifying functionally dependent residues in the HIV protease and other systems [6,5,4,3,7]. For each of the  = 3, 321 pairs of positions, a 2 × 2 contingency table was constructed for both the drug-naive sequences and the sequences treated by two or more drugs. The binomial or "product moment" [11] correlation coefficient

(1)

was calculated for both datasets, where for amino acid position *A *we denote the wild-type state as *A*_0 _and the mutated state as *A*_*m*_. Of the 3, 321 *ϕ *values, 98 from the drug-naive and 223 from the treated set were considered to be statistically significant with substantial correlations (|*ϕ*| > 0.1, *p *< 0.001). These correlations match qualitatively with a prior study which used the same database but had fewer sequences: the *ϕ *values of the top 15 positively correlated pairs for the PI2+ cohort and those of Wu, et al. [3] have a Spearman rank order correlation coefficient of 0.80 (data not shown). Furthermore, we observe larger *ϕ *values for pairs of drug-associated positions compared to non-drug associated pairs (Figure 3), which is consistent with previous observations [3].

Previous studies of pair correlations in protein families have indicated that coevolving pairs of residues tend to lie closer to each other in structure than random residue pairs [27-31]. In the case of the HIV protease, the distribution of *ϕ *values for drug-associated positions with distance shows a characteristic triangular shape, particularly for the PI2+ cohort (Figure 3B) [32]. In particular, distances associated with the most correlated residue pairs (30–88, 54–82, 32–47, and 37–77) are all within a few Ångstroms of each other. The triangular shape in Figure 3B is not surprising, given that most of the drug-associated positions are on the substrate cleft and thus tend to be relatively close to each other in space. As a result, 78 pairs (36% of the statistically significant pairs) are within 8 Å of each other. We also see no tendency for exposed residues to preferentially coevolve, in contrast to previous studies on mutation covariation in other protein families [27,29,15].

Of the 26 statistically significant negatively correlated pairs in the PI2+ cohort, 10 pairs involve either residues 30 or 88. Residue 30 is negatively correlated with positions 82, 10, 46, 90, 54, 73 and 84 (in decreasing order of the magnitude of the correlation), while position 88 is negatively correlated with positions 82, 73 and 54. It is interesting to note that position 63, which has a high mutation rate in both the PI0 and PI2+ cohorts, is negatively correlated with positions 80, 52, 5, 83, 64, and 61, of which only position 83 is associated with drug resistance [3]. In fact, of the 23 unique positions involved in the 26 negatively correlated pairs, most, but not all, are positions of drug resistance. It is possible that the 7 non-drug associated positions, 5, 15, 20, 52, 61, 64 and 80 play a role in the stability or function of the protein, even if they do not interfere with inhibitors [33-35]. Residue 80, in particular is negatively correlated with three residues, 63, 71 and 90, all of which play either primary or accessory roles in drug resistance.

**Figure 4:**
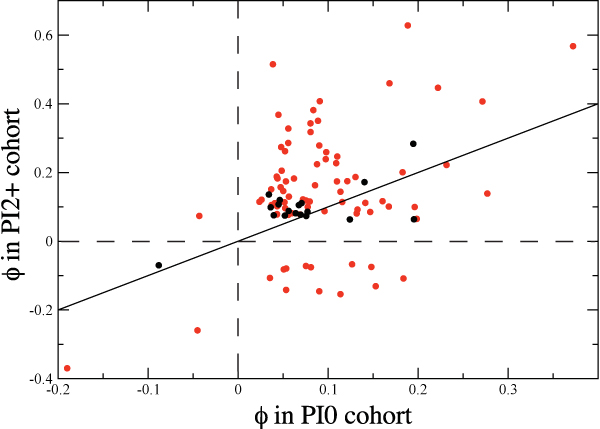
**Scatterplot showing the change in *ϕ *statistic upon drug treatment, with drug associated positions in red and non-drug associated positions in black**. Only pairs with statistically significant (p < 0.001) pair correlations are shown. The solid line corresponds to no change upon drug treatment.

Drug treatment has a significant impact on the pair correlations, as can be seen in Figure 4. Of the  = 3, 321 pairs of positions, only 100 have statistically significant (*p *< 0.001) *ϕ *values and are common to both the PI0 and PI2+ cohorts, and of these, most of the positively correlated pairs in the PI0 cohort become more strongly correlated in the PI2+ cohort. However, some pairs of residues which are weakly positively correlated in the PI0 cohort become negatively correlated in the PI2+ cohort. There are 12 such pairs, almost all of which have at least one primary drug resistance position, and 8 of them involve either residue 30 or 88. It is interesting to note that in PI0 cohort sequences, residue 30 is positively correlated with positions 24, 46, 54, 84 and 90, but becomes strongly negatively correlated in the PI2+ cohort. The anticorrelation of residue 30 with the other primary positions after drug treatment has been previously observed experimentally [36] and in a prior statistical study [3], but it is not clear why this anticorrelation exists only in the presence of drugs.

Additionally, the types of residues involved in pair correlations changes upon treatment, with accessory positions becoming more prominent: 10% of the correlated pairs in the PI0 cohort consist of a primary and an accessory position, and that this combination increases to 31% in the PI2+ cohort. Furthermore, 45% of the pairs in the PI0 cohort involve at least one accessory position, which increases to 61% in the PI2+ cohort. As expected, pairs of non-resistance-associated positions decrease from 52% to 35%. It is interesting to note that the same trend is not observed for primary positions: 57% of pairs in the PI0 cohort contain at least one primary position, and this is essentially unchanged in the PI2+ cohort (59%). Therefore, drug treatment causes correlated pairs involving primary and non-resistance associated positions to be replaced by pairs involving primary and accessory positions.

### Exhaustive analysis of residue triples

To study interactions among mutations beyond the pair level, we quantify the amount of information in the observed distribution that cannot be explained by pair correlation. This is done using the three-body "connected information" [12], which is defined as the difference in Shannon entropy *S*(*P*) = -∑_*i*_*p*_*i *_log *p*_*i *_between the distributions *P*(*A*, *B*, *C*) and (*A*, *B*, *C*), where the latter is the maximum entropy distribution subject to the constraints that all of its univariate and bivariate marginals are the same as that of *P*(*A*, *B*, *C*):

(2)

Similarly, we define the two-body connected information to be

(3)

The total information arising from correlation at any level is given by the "multi-information" or the Kullback-Leibler divergence between the observed distribution and the prediction based on an independent model:

(4)

**Figure 5:**
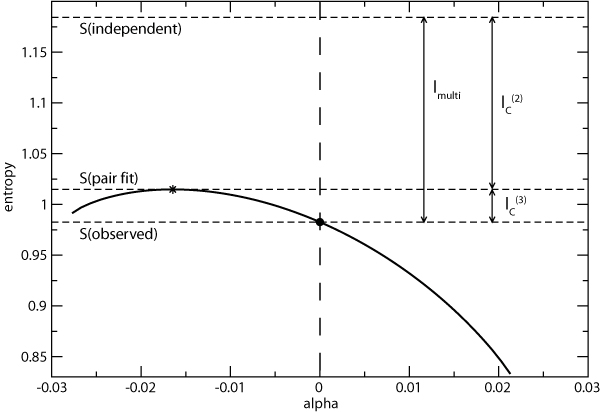
**The Shannon entropy for the family of all triplet distributions having the same univariate and bivariate marginals as the data for the triplet 46-54-90 (PI2+ cohort) plotted as a function of the parameter *α *defined in Equation 8**. The observed distribution is indicated by the filled circle, and the entropies of the independent model (*S*_*ind*_), the model with only pair interactions (*S*_*pair*_) and the observed data (*S*_*obs*_) are indicated, as well as *I*_*multi *_and the connected information measures  and . *S*_*pair *_is the entropy of the distribution denoted by (A, B, C) in the text which has the maximum entropy subject to the constraints that all of the univariate and bivariate marginals are the same as the observed distribution, and is denoted in the figure by a star.

The maximum entropy distribution (*A*, *B*, *C*) can be thought of as being "in between" the independent model and the observed distribution, since it is more constrained than the independent model but does not have the full correlation structure of the observed data (Figure 5). Since , we can interpret the two- and three-body connected information as the part of the correlation that can be explained by pair interactions alone, and that which arises from three-body interactions, respectively [12].

We examined the degree of connected information in both the PI0 and PI2+ cohort sequences. The connected information  was calculated for all  = 88, 560 residue triples in the HIV protease as described in the Methods section below. In the PI0 cohort sequences, only 175 residue triples have statistically significant three-body interaction at the *p *= 0.001 level, while the PI2+ cohort sequences have 6,300 significant triples. Furthermore, the significant triples from the PI2+ cohort are enriched in drug resistance associated positions: 32% of them consist of only drug associated positions, compared to 12% of the full set of 88,560. In contrast, the significant triples from the PI0 cohort show no such enrichment: only 9% consist solely of drug resistance associated positions.

**Figure 6:**
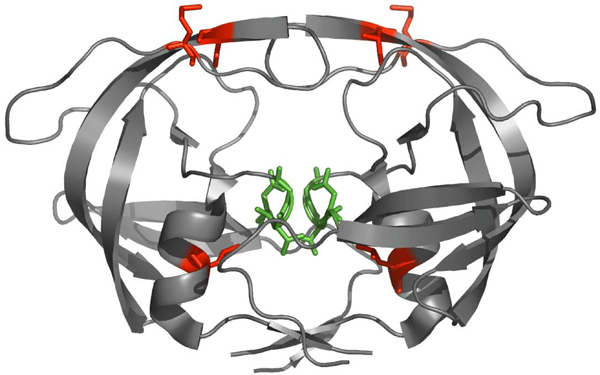
**Structure of the HIV protease dimer **(PDB code 1HN0). The catalytic triad (residues 25, 26, and 27) is highlighted in green, while the residues from the triple with the largest  in the PI2+ cohort (46-54-90, see Figure 9) are shown in red. Residue 90 is close to the catalytic triad, whereas residues 46 and 54 are close to each other, with the smallest distance between heavy atoms of 3.11 Å).

**Figure 7:**
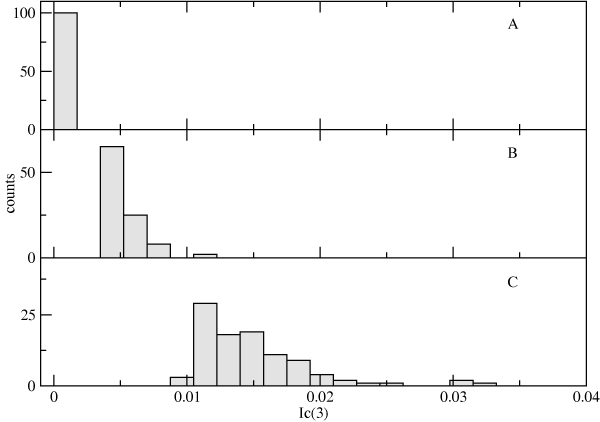
**Distribution of the 100 largest  values for the PI0 (A), PI1 (B), and PI2+ (C) cohorts**.

**Figure 8:**
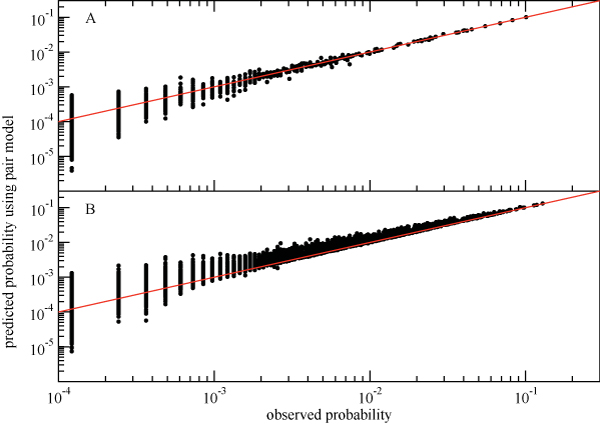
**Predicted vs observed probabilities for a triple mutant for all triples of drug-associated positions in the HIV protease using sequences from the PI0 (A) and PI2+ (B) cohorts**. Mutational states that were unobserved in the database and would have an observed probability maximum likelihood estimate of zero are not shown. The dots correspond to the best fit pair-term model, and the red lines of slope 1 correspond to the perfect agreement of the predicted probabilities with those observed, which would be obtained if the three-body interactions were included.

The 10 triples with the largest  values from the PI2+ cohort sequences are shown in Table 1, along with the corresponding values of *I*_*multi*_. Residues from the largest  triple (46-54-90) are also displayed on the structure of the HIV protease (Figure 6). As can be seen in Table 1, the total contribution of three-body interactions to the information content of the observed data is at most 10%. Although this is a relatively small effect, there is a clear association with drug therapy, with the largest  values increasing substantially with the number of PIs the patient was exposed to (Figure 7). There is relatively little overlap between the largest 100  triples in the PI0 and PI2+ cohorts, with only 11 triples in common. The impact of these higher-order interactions is particularly manifest in the probability of the occurrence of three simultaneous mutations in a given residue triple (Figure 8), which is significantly increased compared to what would be predicted based on a pair model for sequences from the PI2+ cohort (but not in sequences from the PI0 cohort).

**Table 1:** The 10 triples in HIV protease from the PI2+ cohort sequences with the largest  (*A*, *B*, *C*) values (all three-body interactions are significant with *p *≪ 10^-6^).

residue triple	(*A*, *B*, *C*)	*I*_*multi *_(*A*, *B*, *C*)
46-54-90	0.03219	0.20170
46-71-90	0.03101	0.29041
46-82-90	0.03085	0.20640
82-84-90	0.02570	0.13237
10-46-90	0.02445	0.31475
46-71-73	0.02195	0.16610
36-46-90	0.02191	0.15949
46-54-71	0.02072	0.23595
46-77-82	0.02068	0.14573
20-82-90	0.02030	0.14661

**Figure 9:**
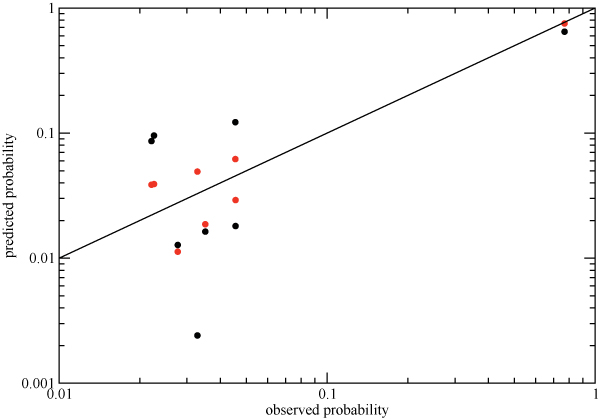
**Predicted vs observed probabilities for the residue triple in HIV protease with the largest  in the PI2+ cohort (46-54-90)**. The black dots correspond to the best-fit independent model, while the red dots correspond to the best-fit pair-term model (Equation 11). The solid line of slope 1 corresponds to perfect agreement of the predicted data with the observed, which would be obtained if the three-body term were included.

While all of the triples in Table 1 have three-body interactions that are highly statistically significant, it is also important to obtain a practical feeling for the magnitude of these interactions. Let us consider the 46-54-90 triple. In Figure 9 we show the correlation between the predicted and observed probabilities for each of the 8 binary states for the independent model (red) and the two-body fit (black) for this triple in the PI2+ cohort. It is clear that the independent model fits the data poorly: the probability that all three residues are mutated is underestimated by an order of magnitude, and some of the others are over- or underestimated by factors of 2 or 3. Although the two-body fit does a better job of reproducing the observed probabilities, it still leads to noticeable deviations from the straight line for the 46-54-90 triple.

Another way in which the magnitude of the effect of the three-body interactions can be visualized is by considering conditional probabilities for the 46-54-90: *P*(54_*m*_|46_*m*_90_*m*_) = 0.4186, *P*(54_*m*_|46_*m*_90_0_) = 0.5508, *P*(54_*m*_|46_0_90_*m*_) = 0.4359, and *P*(54_*m*_|46_0_90_0_) = 0.0280. All of these probabilities differ significantly from the independent estimate of *P*(54_*m*_) = 0.1178. We also see clear evidence of "triplet correlation" in the data, in the sense that the probability of a mutation depends very strongly on the state of both of the other residues, e.g. residue 54 is much less likely to be mutated if *both *46 and 90 are wild-type than if only one of them is wild-type.

Much of this probabilistic dependency, however, can be accounted for by pair interactions. The corresponding probabilities for the best-fit two-body model are (54_*m*_|46_*m*_90_*m*_) = 0.6286, (54_*m*_|46_*m*_90_0_) = 0.2236, (54_*m*_|46_0_90_*m*_) = 0.2316, and (54_*m*_|46_0_90_0_) = 0.0488. Even though this model contains no three-body interactions, a qualitative "triplet correlation" of the type seen above remains (i.e. (54_*m*_|46_*m*_90_0_) ≈ (54_*m*_|46_0_90_*m*_) > (54_*m*_|46_0_90_0_)), indicating that non-trivial three-way probabilistic dependencies can arise purely from pair correlations [37]. Overall, three-body interactions do quantitatively modulate the probabilities, but only to a small degree, since  is an order of magnitude smaller than *I*_*multi *_even for the triples in Table 1. In other words, the contribution of three-body interactions to any of the triplet distributions that describe the mutational patterns of protease taken three at a time is roughly 10% or less of the effect induced by the pairwise interactions acting on these positions. Nonetheless, there can be substantial effects on a "micro level", such as the more than twofold difference in *P*(54_*m*_|46_*m*_90_0_) seen here.

### Increased higher-order interactions in larger residue groups

**Figure 10:**
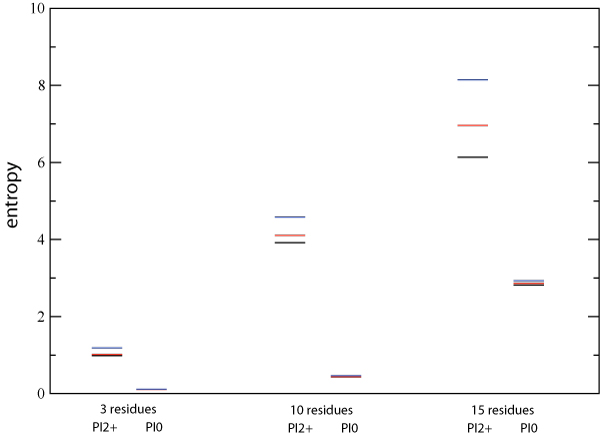
**Shannon theoretic entropy for the observed (black), best pair-term model (red) and independent model (blue) for the three-residue group 46-54-90, the 10-residue group 20-32-46-48-53-54-58-74-82-90, and the 15-residue group 10-20-33-36-46-54-55-63-71-73-74-82-84-90-93 for both the PI0 and PI2+ cohorts**.

The small but consistent systematic deviations seen in Figure 8B raises the possibility that these interactions could combine synergystically to produce more substantial effects over larger clusters of residues. Ideally, this would be studied by fitting log-linear models to increasingly larger clusters using the data from the PI2+ cohort. However, the size of cohort limits our ability to do this to clusters of no more than ≈ 15 residues. We begin by examining the 10-residue group 20-32-46-48-53-54-58-74-82-90, which was chosen to have the largest higher-order interactions from among a limited set of residues defined by the three key primary drug resistance positions 30, 82 and 90, and the known accessory positions associated with them. For this 10-residue group in the PI2+ cohort, we observe strong pair interactions which bring observed and predicted probabilities into qualitative, order-of-magnitude agreement, weaker three-body interactions which further improve the agreement, and very weak four-body and higher interactions which have quantitative impact on a small number of state probabilities (Additional File 2). This can be quantified in terms of connected information: from the entropies of the of the observed, three-body model, pair model, and independent distributions (Figure 10), we find that  = *S*(*ind*) - *S*(*pair*) = 0.4785,  = *S*(*pair*) - *S*(*trip*) = 0.1187, and the sum of the remaining connected information measures of fourth order and higher is *S*(*trip*) - *S*(*obs*) = 0.0703 (these information theoretic measures were found to be robust with respect to sampling error as determined by bootstrap). When compared to *I*_*multi *_= *S*(*ind*) - *S*(*obs*) = 0.6676, we see that three-body and higher-order interactions make up 28% of the total correlation information. This is substantial increase over the (at most) 10% contribution from higher-order interactions to the observed triplet distributions. By contrast, the same 10-residue group for the PI0 cohort displays a substantially smaller overall degree of correlation, as seen by the small *I*_*multi *_in Figure 10.

**Figure 11:**
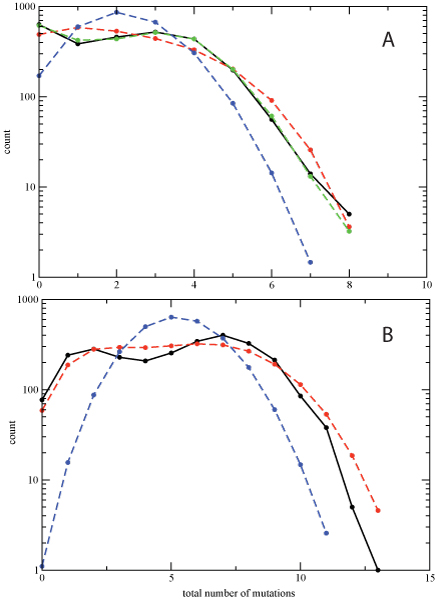
**Total number of observed (solid black curve) or predicted mutations in the 2,702 sequences of the PI2+ cohort for the 10-residue group 20-32-46-48-53-54-58-74-82-90 (A) and the 15-residue group 10-20-33-36-46-54-55-63-71-73-74-82-84-90-93 (B)**. The dashed curves represent the predictions for the independent (blue), pair-term (red), and pair+three-body model (green, not fit for the 15-residue group).

We also studied the distribution of the total number of mutations in the same 10-residue group (20-32-46-48-53-54-58-74-82-90). Appropriate subsets of the state probabilities determined above were summed to obtain the distribution of the total number of mutated residues for the independent, two-body, and three-body models, and the results are shown in Figure 11A. The distribution for the independent model is very different from the observed distribution: the probability of having no mutations is considerably underestimated, and the upper tail is much too thin. Adding pair terms greatly improves the "no mutation" probability and considerably extends the length of the tail. The tail length is further modulated by the addition of the three-body interactions, bringing the distribution very close to the observed probabilities.

It should be noted that since the univariate marginals are preserved by all of the models, the mean total number of mutations is the same for all 4 curves in Figure 11A. Therefore, under- or overestimation of the total number of mutations in one part of the distribution must be compensated by over- or under-estimation (respectively) in another, implying that the curves must cross one other. To quantify this effect, we can compare the predicted probabilities for seeing 5 or more mutations under each model and comparing to the observed probability. Those probabilities are 0.0372, 0.1197, and 0.1030 for the independent, pair-term model, and pair+three-body model, respectively, compared to the observed probability of 0.1007. The deviations of the first two from the observed are highly statistically significant, while the latter has a p-value of ≈ 0.06. The qualitative distribution is well accounted for by the pair interaction model, with the three-body and higher interactions modulating the details of the shape of the distribution, such as the increase in frequency of 3–5 and 13–15 mutations, at the expense of a decrease in the frequency of 6–10 mutations.

To see if the synergistic effects seen for the 10-residue group become even stronger for 15 residues, we repeated this analysis for the 15-residue group 10-20-33-36-46-54-55-63-71-73-74-82-84-90–93 (Figure 10), which was chosen by selecting the residues with the largest change in mutation frequencies upon PI treatment. For the PI2+ cohort, we find that  = *S*(*ind*) - *S*(*pair*) = 1.1810, the sum of the remaining connected information measures of third order and higher is *S*(*pair*) - *S*(*obs*) = 0.8249 and *I*_*multi *_= *S*(*ind*) - *S*(*obs*) = 2.0059. The contribution of three-body and higher-order interactions now make up 41% of the total correlation information. However, this result may somewhat overestimate the true amount of higher-order correlation. A fit with a three-body model (Equation 12) gives an estimate of  of 0.2638, and the ratio  represents a lower bound on the contribution from correlations beyond the pair level. A more complete account of the many issues involved in estimating the amount of higher order correlation and its precision and accuracy for finite data sets will be the subject of a future communication. The comparison of the predicted and observed distributions of total number of mutations bears out this result (Figure 11B), showing more pronounced differences between the observed (black) and pair-model (red) distributions. In addition to the overestimation of the upper tail similar to that seen for the 10-residue group, we now also see that the pair model cannot reproduce the bimodal shape seen in the observed data. Again, the same 15-residue group for the PI0 cohort shows considerably weaker overall correlation (Figure 10).

## Discussion and conclusion

Treating HIV protease with drugs results in the appearance of complex mutational patterns: observed mutations are not limited to the active site and often occur in groups that involve two or more residues. Furthermore, some mutations occur even in the absence of drugs, presumably following neutral rather than adaptive evolution. To study correlations between different residue positions in HIV protease, we have developed a hierarchy of models that allows us to include inter-residue correlations of arbitrary order within a consistent framework. Using only HIV protease sequences as input, we find that pair interactions become common and quite strong after PI treatment. In fact, it is often impossible to achieve even qualitative agreement with the data without including the two-body terms (Figures 9, 11, and Additional File 2). This finding calls into question a common assumption employed in current probabilistic approaches to phylogeny [38] that most residues evolve independently.

We have developed an information-theoretic method to study interactions between mutations beyond the pairwise level. Our approach is based on the notion of the connected information  (Equation 2) [12]. While there are a variety of quantitatively different measures of pair correlation [11,39] that may differ in their sensitivity in various regimes, they all measure essentially the same qualitative feature of the observed data. On the other hand, no single summary statistic can capture all of the various characteristics of higher-order behavior, leading to multiple descriptions that provide complimentary information. Connected information is one intuitive statistic that provides insight into the degree of structure in the data beyond the pair correlation level.

Connected information provides information which is complementary to Bayesian network analyses based on factorizations of the joint probability. It can readily verified that  = 0 if at least one of the random variables is independent of the other two, or if the joint distribution involves conditional independence (e.g. *P*(*A*, *B*, *C*) = *P*(*A*)*P*(*B*|*A*)*P*(*C*|*A*)). However, a joint distribution with triplet-level probabilistic dependencies (in the sense that two of the variables are independent of each other, but the third depends jointly on the state of the other two, e.g. *P*(*A*, *B*, *C*) = *P*(*A*)*P*(*B*)*P*(*C*|*A*, *B*), or if *P*(*A*, *B*, *C*) cannot be factorized into any simpler form) could still be consistent with no three-body connected information if the observed triplet distribution is the maximum entropy distribution relative to its marginals. Thus, even if a Bayesian network-style analysis shows that a given triple cannot be factorized into any simpler form, that "triplet correlation" could still be consistent with a very small or zero , indicating that the observed behavior is dominated by two-body interactions. In fact, it has been shown that very complex correlation patterns among random variables can arise from large numbers of weak pairwise interactions [37].

Other information-theoretic measures of "higher-order correlation" have also been proposed, including higher-order mutual information, which measures "frustration" or the degree of synergy vs redundancy among several random variables [40]. While this measure has been used in the analysis of HIV envelope protein sequence data [41], its interpretation is considerably less intuitive. Similarly, *ad hoc *methods for finding putative clusters of mutually correlated residues [3,9,8,10] cannot reliably uncover sets that have intrinsic higher order interactions, as defined by large  (data not shown).

Plotting *ϕ *values for pairs of residues as a function of the distance between them (Figure 3B) reveals that while some large pair correlations arise from direct contacts between residues (e.g. *ϕ *≈ 0.8, *d *< 5 Å), there are also strong correlations (*ϕ *≈ 0.5) between amino acids separated by 15 Å or more, making physical coupling between them very unlikely. To provide an example of the former, we consider mutations involving residues 30 and 88. The closest distance between heavy atoms of residues 30 and 88 is just 3.66 Å, making likely some sort of physical interaction between them. Mutations at residue 30 are strongly and uniquely associated with resistance to the protease inhibitor nelfinavir, and there exists a strong correlation between mutations at positions 30 and 88 [42] which may be due in part to a compensation of the loss of a surface negative charge from the D30N mutation being restored by N88D [9].

It is possible that chains of intermediate interactions result in long-range coupling between two coevolving yet physically distant residues [43]. However, because non-zero values of *λ*_*ij *_indicate a presence of *direct *interactions between residues *i *and *j *in our model [15], we can decompose such "energetically connected pathways" into contributions from separate pairs. In contrast to a previous study [15], we find that non-zero values of *λ*_*ij *_are only weakly correlated with distance (Additional File 3). This lack of correlation is not entirely surprising, since even direct interactions between a pair of residues need not have a purely physical origin. Indeed, if protein fitness is a non-linear function of its stability or enzymatic activity [44], two mutations can be correlated because they compensate each other by making independent and opposite contributions to the overall fitness, even if there is no direct or indirect physical interaction between them [45]. By the same argument, the three-body terms also result from a mixture of physical and epistatic (compensatory) origins.

We have shown that three-body and higher-order correlations have the largest effect on the probabilities of the simultaneous occurrence of multiple mutations in the HIV protease (Figure 11). Since both this and previous studies have found that the total number of mutated positions is correlated with treatment by multiple protease inhibitors (Figure 1) [3], the presence of higher-order interactions may influence how protease reacts to multiple drugs, and could have an important impact on the evolution of cross-resistance, for example, by providing the virus with an "escape hatch" of large numbers of mutations. Higher-order interactions could also impact the time evolution of mutations by allowing the virus to pass through otherwise unlikely mutational states. We have seen that the impact of higher-order interactions in 10 to 15 residue clusters is at least a factor of two larger than the largest  values for residue triples (approximately 20% or more of the total entropy change). One of the outstanding questions raised by this work is whether the impact of higher-order interactions for HIV evolving under the pressure of multiple drugs continues to become stronger for larger residue groups (ultimately the set of all 41 drug-associated positions). Unfortunately, there is not enough sequence data to perform such an analysis. Short of obtaining additional data, it may also be possible to explore this question by constructing synthetic data sets using *λ*_*i *_and *λ*_*ij *_values consistent with an observed  distribution at the level of residue triples (i.e. Figure 7C).

The sequence-based approach presented here is not limited to the HIV protease and its response to drug treatment, and should be equally useful in studies of the evolution of drug resistance in other systems. Moreover, it will be of interest to extend our techniques to other examples of short-term neutral and adaptive evolution, including controlled evolution in the lab accompanied by protein sequencing at different timepoints. Recent work has suggested that evolutionary pathways of proteins are relatively restricted and may be predictable in general [46], and specific methods for predicting the mutational dynamics of HIV protease have been proposed, based on Bayesian network models [47] or pairwise conditional selection pressure [5]. A better understanding of the nature of the probabilistic dependencies underlying the network models should lead to improved prediction strategies. However, our model cannot distinguish between physical and epistatic origins of the observed co-evolution. To do this, we need a different approach which would explicitly introduce protein fitness as a function of residue energies (including interactions across protein-protein and protein-ligand interface). These energies would be fit against the sequence data, resulting in a prediction that decomposes observed inter-residue correlations into the physical and epistatic parts. This approach is currently being pursued in our laboratories.

## Methods

### HIV sequence database

Aligned and annotated HIV-1 protease amino acid sequences were obtained using the web interface of the Stanford HIV Drug Resistance Database [16]. The sequences are all classified under the HIV-1 Main group, subtype B. For the purposes of this paper, we are considering only the number of protease inhibitors and not the specific combinations of protease inhibitors used in the treatment. There are 13,608 sequences in this curated set, of which 8,229 sequences are drug naive, 2,677 sequence are associated with PI monotherapy, and the remaining 2,702 sequences are associated with between 2 through 6 protease inhibitors.

### Calculation of higher-order interactions

For residue triples, we compute  by writing the triplet probability distribution in log-linear form [11]:

(5)

where we assign numerical values to the states of *A*, *B *and *C*, e.g. 0 and 1. We find (*A*, *B*, *C*) by setting *λ*_*ABC *_= 0 in Equation 5 and fitting the six parameters *λ*_*i *_and *λ*_*ij *_to the values that maximize the likelihood of the data under a multinomial model [48]. However, in the triplet case it is possible to avoid direct nonlinear optimization: let us represent the 8 observed probabilities by the vector

(6)

where, e.g. *p*_0*m*0 _= *P*(*A*_0_, *B*_*m*_, *C*_0_). It is sufficient to consider only the three marginals *P*(*A*_*m*_), *P*(*B*_*m*_), and *P*(*C*_*m*_), and three suitably chosen bivariate marginals, e.g. *P*(*A*_*m*_, *B*_*m*_), *P*(*A*_*m*_, *C*_*m*_), and *P*(*B*_*m*_, *C*_*m*_), since the remaining 9 bivariate marginals can be reconstructed as combinations of these: *P*(*A*_*m*_, *B*_0_) = *P*(*A*_*m*_) - *P*(*A*_*m*_, *B*_*m*_), *P*(*A*_0_, *B*_*m*_) = *P*(*B*_*m*_) - *P*(*A*_*m*_, *B*_*m*_), *P*(*A*_0_, *B*_0_) = 1 - *P*(*A*_*m*_) - *P*(*B*_*m*_) + *P*(*A*_*m*_, *B*_*m*_), etc. The six marginals can then be written as a matrix equation involving **p**_0_:

(7)

Since the matrix multiplying **p**_0 _is rectangular with dimensions 6 × 8, it has a two-dimensional null space, with basis vectors **n**_1 _= (1, 0, 0, 0, 0, 0, 0, 0) and **n**_2 _= (0, -1, -1, 1, -1, 1, 1, -1). Then, any linear combination *α*_1_**n**_1 _+ *α*_2_**n**_2 _added to **p**_0 _will not change the marginals. However, *α*_1 _and *α*_2 _cannot be chosen independently without violating the normalization of **p**_0_: we must choose *α*_1 _= *α*_2 _= *α*. Therefore, the family of all possible distributions that have the same univariate and bivariate marginals are mapped out by the parameter *α *using the relation

(8)

where the feasible values of *α *are constrained by the non-negativity requirement for probabilities. Furthermore, *λ*_*ABC *_= 0 in Equation 5 implies that

(9)

Therefore, to find (*A*, *B*, *C*) it suffices to find the value of *α *which satisfies Equation 9:

(10)

leading to a cubic equation in *α *[11].

To obtain the maximum entropy distributions for more than three binary random variables, nonlinear optimization is unavoidable. However, instead of directly maximizing the entropy subject to the marginal probability constraints, we maximize the likelihood subject to the constraints that the *λ*'s vanish beyond a given order [48]. In most cases, the latter will be more computationally efficient since the number of *λ *variables grows polynomially with the number of variables, while the dimensionality of the null space defined by the marginal probability constraints (which is one-dimensional for three variables) increases exponentially with the number of variables.

In general, we fit data on mutation and wild-type amino acid counts to the following hierarchy of probabilistic models: the independent model *P*(*A*, *B*, *C*,...) = *P*(*A*)*P*(*B*)*P*(*C*)⋯, the "two-body" model:

(11)

and the "three-body" model:

(12)

where *λ *is the vector of parameters, the indices (*i*, *j*) and (*i*, *j*, *k*) run over all distinct combinations of {*A*, *B*, *C*,...} with *i *≠ *j *and *i *≠ *j *≠ *k*, respectively, and *I*, *J*, and *K *are numerical values of the corresponding random variable (we use 0 for wild-type and 1 for mutant). For an *n*-variate distribution *P*(*A*, *B*, *C*,...) there are *n*(*n *- 1)/2 pair parameters *λ*_*ij *_and *n*(*n *- 1)(*n *- 2)/6 three-body parameters *λ*_*ijk *_(*λ*_0 _is a normalization constant). The independent model was determined by forming products of the observed univariate marginals. The magnitudes of the *λ*_*i *_and *λ*_*ij *_parameters in the two-body model are related to the mutation frequencies at site *i *and pair correlations between sites *i *and *j*, respectively. In fact, the magnitudes of *λ*_*ij *_in the context of a two-body model have been proposed as a measure of "direct information", i.e. the part of pair correlation resulting from direct coupling [15]. It should be noted that the relative magnitudes of *λ*_*ij *_is dependent on the choice of the numerical values assigned to the random variables *I*, *J *and *K *in Equations 11 and 12. In this work, we assign values of 0 and 1 for wild-type and mutant, respectively, for computational convenience. It has been argued, however, that a more appropriate choice of numerical values is one which is symmetric about zero, e.g. ± 1, which allows "gauge constraints" to be introduced [15]. While this choice will affect the the values of *λ*_*ij *_and their interpretation as "direct information", it will not change the best-fit two-body or three-body probabilities and consequently will have no impact on the values of  and related measures of higher-order interactions.

The two-body model for *n *= 3 was fit by solving Equation 10 exactly [49]. If no feasible solution of Equation 10 exists, then  was set to zero. For *n *≥ 4, the unknown parameters in Equations 11 and 12 were determined by maximizing the multinomial log-likelihood

(13)

where *i *is one of the 2^*n *^states, *N*_*i *_is the number of times that state was observed, and *P*(*i*|*λ*) is the predicted probability for state *i *to be observed given the vector of parameters *λ*. Maximization was performed numerically using the "nlm" function of the R software package [50]. All entropy and connected information values are given in natural log units. Statistical significance of the three-body interactions was estimated using the likelihood ratio test under the null hypothesis that the data were generated by (*A*, *B*, *C*) by Monte Carlo sampling. For all of the residue triples in Table 1, the *p*-values for the observed likelihood ratio were too small to be estimated (*p *≪ 10^-6^), indicating very strong statistical significance for the three-body interaction.

## Competing interests

The authors declare that they have no competing interests.

## Authors' contributions

OH and MA wrote software and performed the analyses, and OH, RML, AVM and MA wrote the paper.

## Supplementary Material

Additional File 1 **Mutation frequencies for selected positions in the HIV protease as a function of the number of PIs the patient was exposed to**. Data is shown for residues 10 (black, solid line), 54 (red, solid line), 90 (green), 71 (blue), 46 (orange), 77 (black, dashed line), and 35 (red, dashed line). Click here for file

Additional File 2 **Predicted vs observed probabilities for the 2_10 _mutational states of the ten-residue group 20-32-46-48-53-54-58-74-82-90 in HIV protease for the PI2+ cohort**. Mutational states that were unobserved in the database and would have an observed probability maximum likelihood estimate of zero are not shown. The black dots correspond to the best-fit independent model, the red dots correspond to the best-fit pair-term model (Equation 11), and the green dots correspond to the best-fit three-body model (Equation 12). The solid line of slope 1 corresponds to perfect agreement of the predicted data with the observed, which would be obtained if all higher-order terms were included. Click here for file

Additional File 3 **Scatterplot of distance vs *λ*_*ij *_parameters estimated using *I *and *J *values of ± 1 as described in the Methods for the 15-residue group 10-20-33-36-46-54-55-63-71-73-74-82-84-90-93 in the PI2+ cohort**. The value along the *y*-axis is the closest distance between any two heavy atoms of the two residues based on the crystal structure of wild type protease (PDB ID 1PRO). Click here for file
