# Supplementary material for: Pairwise and higher-order correlations among drug-resistance mutations in HIV-1 subtype B protease
Source: BMC Bioinformatics. 2009 Aug 27;10(Suppl 8):S10. doi: 10.1186/1471-2105-10-S8-S10 (PMC2745583; doi:10.1186/1471-2105-10-S8-S10)
Supplement: Additional File 1 — Mutation frequencies for selected positions in the HIV protease as a function of the number of PIs the patient was exposed to. Data is shown for residues 10 (black, solid line), 54 (red, solid line), 90 (green), 71 (blue), 46 (orange), 77 (black, dashed line), and 35 (red, dashed line). [file 1471-2105-10-S8-S10-S1.pdf]

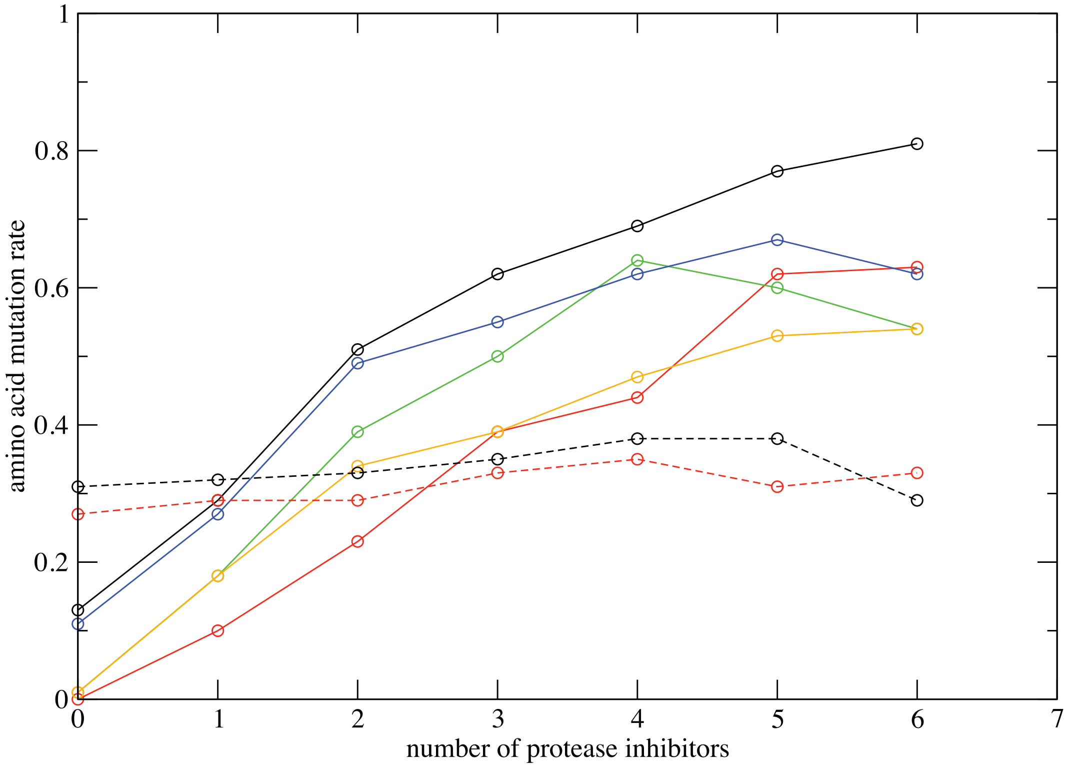

Additional File 1: Mutation frequencies for selected positions in the HIV protease as a function of the number of PIs the patient was exposed to. Data is shown for residues 10 (black, solid line), 54 (red, solid line), 90 (green), 71 (blue), 46 (orange), 77 (black, dashed line), and 35 (red, dashed line).
