# Supplementary material for: Pairwise and higher-order correlations among drug-resistance mutations in HIV-1 subtype B protease
Source: BMC Bioinformatics. 2009 Aug 27;10(Suppl 8):S10. doi: 10.1186/1471-2105-10-S8-S10 (PMC2745583; doi:10.1186/1471-2105-10-S8-S10)
Supplement: Additional File 2 — Predicted vs observed probabilities for the 210 mutational states of the ten-residue group 20-32-46-48-53-54-58-74-82-90 in HIV protease for the PI2+ cohort. Mutational states that were unobserved in the database and would have an observed probability maximum likelihood estimate of zero are not shown. The black dots correspond to the best-fit independent model, the red dots correspond to the best-fit pair-term model (Equation 11), and the green dots correspond to the best-fit three-body model (Equation 12). The solid line of slope 1 corresponds to perfect agreement of the predicted data with the observed, which would be obtained if all higher-order terms were included. [file 1471-2105-10-S8-S10-S2.pdf]

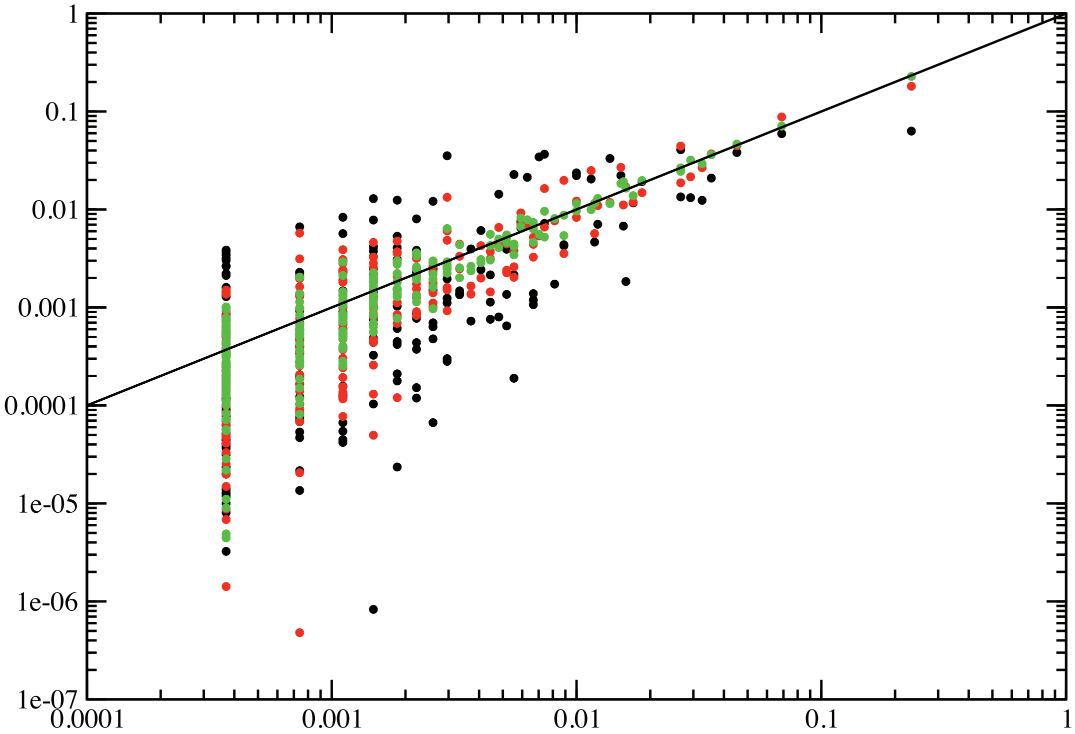

Additional File 2: Predicted vs observed probabilities for the 2^{10} mutational states of the ten-residue group 20–32–46–48–53–54–58–74–82–90 in HIV protease for the PI2+ cohort. Mutational states that were unobserved in the database and would have an observed probability maximum likelihood estimate of zero are not shown. The black dots correspond to the best-fit independent model, the red dots correspond to the best-fit pair-term model (Equation 11), and the green dots correspond to the best-fit three-body model (Equation 12). The solid line of slope 1 corresponds to perfect agreement of the predicted data with the observed, which would be obtained if all higher-order terms were included.
