# Supplementary material for: Pairwise and higher-order correlations among drug-resistance mutations in HIV-1 subtype B protease
Source: BMC Bioinformatics. 2009 Aug 27;10(Suppl 8):S10. doi: 10.1186/1471-2105-10-S8-S10 (PMC2745583; doi:10.1186/1471-2105-10-S8-S10)
Supplement: Additional File 3 — Scatterplot of distance vs λij parameters estimated using I and J values of ± 1 as described in the Methods for the 15-residue group 10-20-33-36-46-54-55-63-71-73-74-82-84-90-93 in the PI2+ cohort. The value along the y-axis is the closest distance between any two heavy atoms of the two residues based on the crystal structure of wild type protease (PDB ID 1PRO). [file 1471-2105-10-S8-S10-S3.pdf]

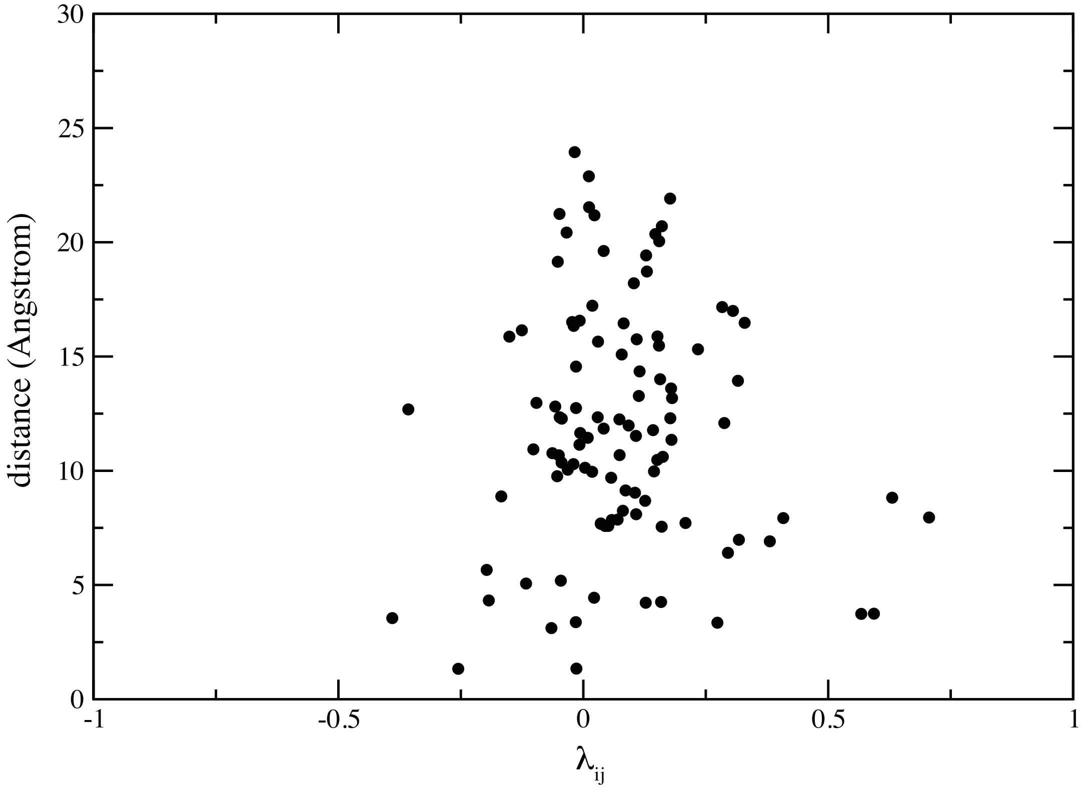

Additional File 3: Scatterplot of distance vs λ_{ij} parameters estimated using I and J values of ± 1 as described in the Methods for the 15-residue group 10–20–33–36–46–54–55–63–71–73–74–82–84–90–93 in the PI2+ cohort. The value along the y-axis is the closest distance between any two heavy atoms of the two residues based on the crystal structure of wild type protease (PDB ID 1PRO).
